# Unusual histological findings after partial pancreaticoduodenectomy including benign multicystic mesothelioma, adenomyoma of the ampulla of Vater, and undifferentiated carcinoma, sarcomatoid variant: a case series

**DOI:** 10.1186/1752-1947-4-402

**Published:** 2010-12-10

**Authors:** Nadja Lehwald, Kenko Cupisti, Stephan E Baldus, Patric Kröpil, Jan Schulte am Esch, Claus F Eisenberger, Wolfram T Knoefel

**Affiliations:** 1 Department of General, Visceral and Pediatric Surgery; Heinrich-Heine-University Duesseldorf, Moorenstr. 5, 40225 Duesseldorf, Germany; 2 Department of Pathology; Heinrich-Heine-University Duesseldorf, Moorenstr. 5, 40225 Duesseldorf, Germany; 3 Department of Radiology; Heinrich-Heine-University Duesseldorf, Moorenstr. 5, 40225 Duesseldorf, Germany

## Abstract

**Introduction:**

The standard operation for carcinoma of the pancreatic head is a partial pancreaticoduodenectomy. Unusual histological findings may occasionally occur in the surgical specimen. We present three unusual histologic diagnoses after pancreaticoduodenectomy.

**Case presentations:**

In the first case, an 86-year-old Caucasian woman was admitted with abdominal pain and nausea. Preoperative evaluation showed a 3 cm cystic lesion in the head of the pancreas. Pathology revealed a benign multicystic mesothelioma. In the second case, a 45-year-old Caucasian man complained of nausea, vomiting and general malaise for several months. Endoscopic retrograde cholangiopancreatographic examination and a computed tomography scan showed a stenosis of the distal bile duct secondary to a mass in the head of the pancreas and duodenum. Histology showed an adenomyoma of the ampulla. In the third case, a 59-year-old Caucasian man presented with chronic alcoholic pancreatitis. A computed tomography scan revealed a 3.5 cm lesion in the head of the pancreas with cystic and solid components. Pathology showed an undifferentiated carcinoma, sarcomatoid variant.

**Conclusion:**

Partial pancreaticoduodenectomy is usually performed for ductal adenocarcinomas, neuroendocrine tumors or chronic pancreatitis. Compared to the majority of the above diagnoses, the three cases in our study are very rare. Benign multicystic mesothelioma is a very rare tumor that originates from the peritoneum. Although it demonstrates a benign clinical behaviour, it frequently recurs after resection. Adenomyoma of the bile duct or ampullary region is a very unusual, benign, localized lesion characterized by adenomyomatous hyperplasia. Undifferentiated carcinoma, sarcomatoid variant, is an aggressive tumor and is characterized by spindle cells. As the lesions were suspicious for carcinoma, partial pancreaticoduodenectomy was justified in all three patients. The histologic diagnosis after partial pancreaticoduodenectomy may differ from the preoperative and intraoperative findings. These cases demonstrate that a definitive diagnosis may only be obtained by a pathologic examination of the surgical specimen.

## Introduction

The standard operation for pancreatic cancer within the head of the pancreas or uncinate process is a partial pancreaticoduodenectomy ('Whipple procedure'). The standard Whipple procedure involves the removal of the head of the pancreas, the distal part of the stomach, the duodenum, the first part of the jejunum, the common bile duct and the gallbladder. Pancreaticoduodenectomy was popularized by Dr Allen Whipple after his success in his initial three cases in 1935 [1]. The technique has undergone several technical modifications and revisions, so that morbidity and mortality rates have dramatically decreased over the past several decades [2]. In 1978, Traverso and Longmire described the pylorus-preserving pancreaticoduodenectomy (PPPD), a procedure already mentioned by Watson in 1944 [3]. The expected advantages of this procedure were less dumping, improved gastrointestinal function and reduced jejunal ulceration.

Occasionally, unusual pathology was present in the surgical specimens which were not initially suspected. We present three unusual histological findings.

## Case presentations

### Case 1

**Figure 1:**
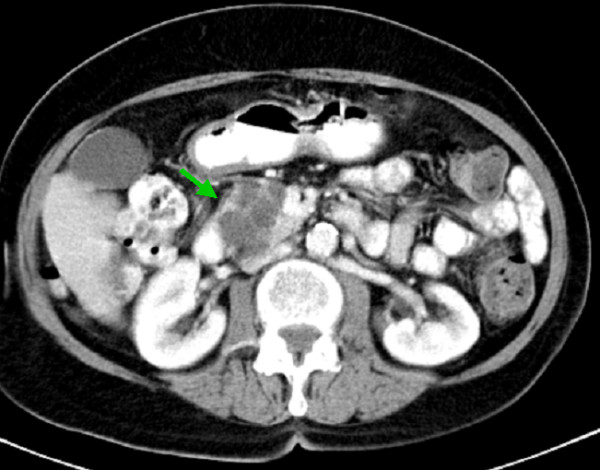
**Benign multicystic mesothelioma of the head of the pancreas: computed tomography scan of the abdomen shows a multicystic lesion of the head of the pancreas with dilated pancreatic duct and regional lymphadenopathy**.

An 86-year-old Caucasian woman was admitted with nausea and abdominal pain. Abdominal and endoscopic ultrasound, as well as a computed tomography (CT) scan, showed a 3 cm cystic lesion in the head of the pancreas with a dilated pancreatic duct and regional lymphadenopathy (Figure 1). Ultrasound-guided fine needle aspiration showed no malignant cells. Preoperative CA19-9 level was 54.1 U/mL (reference value < 27 U/mL). Nevertheless, based upon the morphological and radiological findings, a malignant cystic pancreatic tumor (for example, cystadenocarcinoma) was suspected. Intraoperatively, a firm, cystic lesion was found in the head of the pancreas with suspicious infiltration of the superior mesenteric vein (SMV). We were unable to exclude malignancy on frozen section, so we performed a modification on Whipple procedure with a partial resection and end-to-end anastomosis of the SMV. The postoperative course was uneventful. Ten months later, the patient had no signs of recurrence.

**Figure 2:**
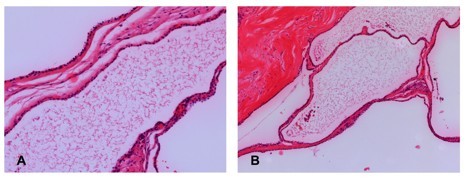
**Histologic findings of benign multicystic mesothelioma of the pancreas**. (A) The tumor is composed of thin-walled, multilocular cysts (100×). (B) The wall of the cyst, lined with single layer of cuboidal epithelium and separated by thin collagenous septae (200×).

A gross 2.5 cm tumor of the head of the pancreas was described. Histopathology showed multiple small cysts covered by cubic mesothelial cells with uniform, small nuclei (Figure 2A and 2B). Immunohistochemistry revealed a strong immunoreactivity for cytokeratin, vimentin and CK5. CD31 and CD34 immunoreactivity was not expressed by the cystic epithelium. There was no invasive growth or malignancy. Based on the histopathological and immunohistochemical findings, the final diagnosis was benign multicystic mesothelioma.

### Case 2

**Figure 3:**
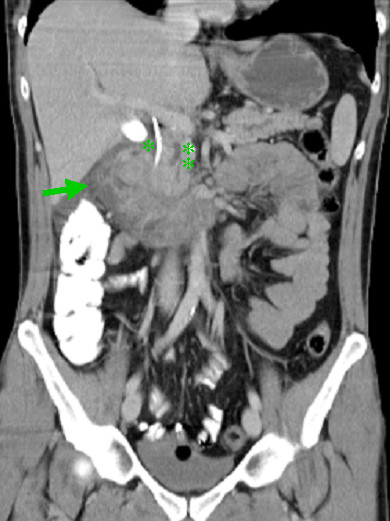
Adenomyoma of the pancreas: abdominal computed tomography scan reveals severe duodenitis (↦), a stent in the common hepatic duct (*) and a dilated duct of Wirsung (**).

A 45-year-old Caucasian man complained of nausea, vomiting and general malaise for several months. CA19-9 was within normal range (20.5 U/mL). A CT scan of the abdomen and endoscopic retrograde cholangiopancreatographic (ERCP) examination showed a mass in the head of the pancreas and duodenum with a stenosis of the duodenum and distal bile duct (Figure 3). Due to the duodenal stenosis and the recent onset of chronic pancreatitis we performed a duodenum-preserving head of the pancreas resection. There was severe chronic inflammation of the head of the pancreas and body intraoperatively. There was no evidence of peritoneal dissemination or hepatic metastases. An intraoperative frozen section showed atypical cells in the resection margin which could not be clearly identified as benign. Thus, the operation was extended to a pancreaticoduodenectomy. The patient recovered and was discharged on postoperative day (POD) 13.

**Figure 4:**
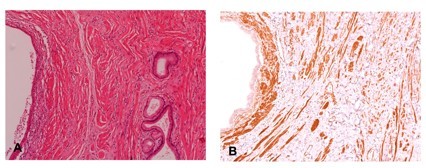
**Histologic findings and immunohistochemical staining for smooth muscle actin in adenomyoma**. (A) Several glands lined by bland looking, single layer of cuboidal to columnar epithelium are surrounded by hyperplastic mesenchymal tissue (100×). (B) Proliferating smooth muscle cells show immunoreactivity for smooth muscle actin.

A macroscopic examination revealed a 5.5 cm lesion in the ampulla of Vater. Microscopically, multiple hyperplastic glands and cysts were found. They were covered by a single-layer epithelium, consisting of cuboidal, columnar and, in part, mucinous cells. Hyperplastic glandular lobes were surrounded by hyperplastic mesenchymal tissue which consisted of muscle fibers, fibroblasts and myofibroblasts (Figure 4A and 4B). There was no invasive component. The lesion was described as an adenomyoma of the ampulla.

### Case 3

**Figure 5:**
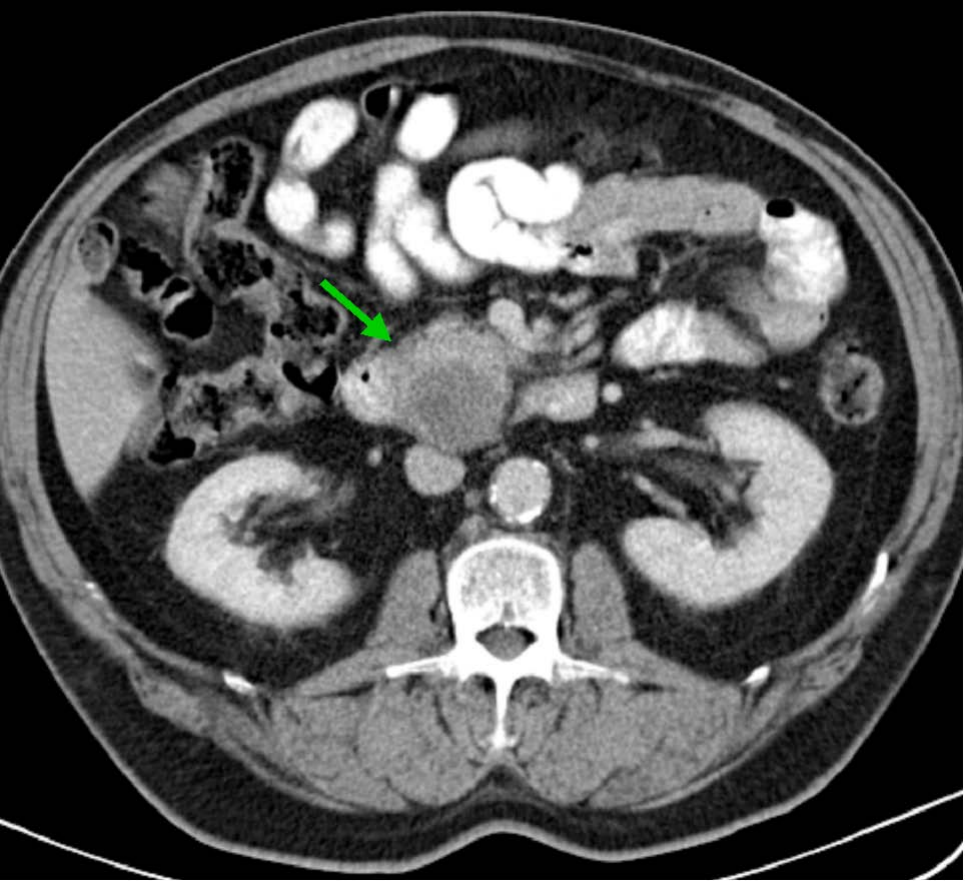
**Undifferentiated carcinoma, sarcomatoid variant of the head of the pancreas: computed tomography scan of the abdomen shows a lesion of the head of the pancreas head with cystic and solid parts**.

A 59-year-old Caucasian man presented with chronic alcoholic pancreatitis. An ultrasound and a CT scan of the abdomen showed a 3.5 cm lesion of the head of the pancreas with cystic and solid parts (Figure 5). The tumor marker CA19-9 was strongly elevated (74.3 U/mL). Due to our suspicion of pancreatic carcinoma on a background of chronic pancreatitis, we performed a partial pancreaticoduodenectomy with radical lymphadenectomy. The postoperative course was uneventful and the patient was discharged on POD 13. The patient was given chemotherapy with gemcitabine according to the protocol for adenocarcinoma following surgery. On a follow-up CT scan eight months after surgery two new liver metastases were discovered. As the patient was in good health, we resected the two liver metastases by the left hemihepatectomy. The patient tolerated the second operation well.

**Figure 6:**
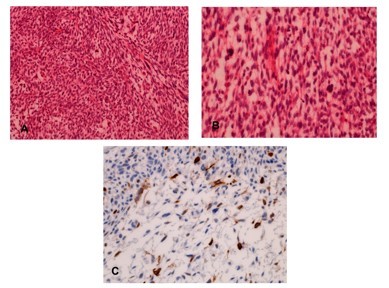
**Histologic findings and immunohistochemical staining for cytokeratin in undifferentiated carcinoma, sarcomatoid variant**. (A) The tumor is highly cellular and arranged in short fascicles and whorls (200×). (B) Tumor cells have oval to spindle shaped hyperchromatic nuclei (400×). (C) Scattered tumor cells are positive for cytokeratin immunostaining (400×).

Macroscopically, a 3.7 cm cystic malignant tumor of the head of the pancreas was found. Microscopically, the malignant tumor contained undifferentiated spindle cells (Figure 6A and 6B). All lymph nodes and resection margins were tumor free. Immunohistochemistry stains were strongly positive for vimentin. Some cells expressed cytokeratin, CK5, desmin and myogenine (Figure 6C). The proliferation index was 30%-40%. Tumor cells were negative for CD34, c-kit, sm-actin and S100. In conclusion, the diagnosis of an undifferentiated carcinoma, sarcomatoid variant was established. The Union for International Cancer Control classification was pT2, pN0 (0/12), pM0, G4 and R0.

## Discussion

The most common indications for pancreatic resection are ductal adenocarcinoma, a neuroendocrine tumor or chronic pancreatitis. The three cases we have described are rare. We would like to present an overview of these unusual conditions:

Benign multicystic mesothelioma was first described by Mennemeyer and Smith in 1979 [4]. It is a very rare tumor originating from the peritoneum but it can also be found on serosal surfaces (for example, intestine, liver and kidney). To our knowledge, this is the first description in the literature of benign multicystic mesothelioma of the pancreas.

Although it is a tumor with a benign clinical behaviour, it frequently recurs after surgical resection. A report of 15 cases revealed a recurrence rate of 26.7% [5,6]. Time between recurrences may range from four months to 12 years [7]. As it has a tendency to recur, a neoplastic etiology is supported. In our patient, 10 months postoperatively there had been no evidence of recurrence.

Benign multicystic mesothelioma is found mainly in women of reproductive age with a past history of abdominal surgery or endometriosis [8]. Only a few reports exist of this gynecologic diagnosis in elderly women [9]. Our patient had not undergone any previous abdominal surgery.

The clinical findings of benign multicystic mesotheliomas are non-specific and include nausea, vomiting and abdominal pain. There is often a palpable abdominal mass, although this was not present in our case [10]. Preoperative diagnosis is often difficult and several differential diagnoses must be considered. As this tumor has a high risk of recurrence, a close follow-up should be performed in every patient.

Adenomyomas are common in the stomach, gallbladder and jejunum but are very unusual in the common bile duct or the ampullary region [11]. Adenomyomas of the extrahepatic bile duct are defined as non-neoplastic, tumor-like localized lesions characterized by glandular and myomatous hyperplasia without cellular atypia [12]. In the absence of gallstones, extrahepatic bile duct adenomyomas often stay dormant for long periods and may eventually cause vague symptoms due to local pressure [13]. They also cause obstructive jaundice, as in this case, which can easily be misinterpreted as a malignant neoplasm. It is difficult to differentiate between biliary adenomyomas and other malignant lesions by radiology (CT). Since malignancy is often suspected preoperatively, surgery with the intent to cure should be performed.

The etiology of adenomyomas of the extrahepatic bile duct and the ampulla of Vater is unknown. As different terms are used in the literature to describe the same histological lesion, the true incidence of adenomyomas of the ampullary region is unclear. In the literature, 30 cases of adenomyomas of the ampulla of Vater and 13 cases of the extrahepatic bile duct have been reported [11,13]. Adenomyomas are usually diagnosed by histopathologic examination after surgery. As in our case, it often turns out to be a surprising diagnosis.

Undifferentiated carcinoma, sarcomatoid variant, is a rare variant of pancreatic tumors. The malignant tumor arises *de novo *or occurs in association with mucinous cysts or other pancreatic neoplasms. Men are affected more often than women with a ratio of 3:1 and a mean age at diagnosis of 63 years. The most common symptoms are fatigue, nausea and vomiting, weight loss and abdominal pain. As in our case, radiologic imaging often shows an aggressive pancreatic mass which is not specific for undifferentiated carcinoma. Undifferentiated carcinomas are usually very large, aggressive neoplasms (average size 9-10 cm size). The prognosis is often limited to several months after resection [14]. Our patient remains in good physical condition seven months postoperatively, although he did develop two new liver metastases under chemotherapy which were resected. In contrast to our case, metastases are usually present at the time of diagnosis, the most common sites being lymph nodes, liver and lung. Surgical resection is the treatment of choice, although most of these neoplasms have already metastasized in the majority of patients by the time of diagnosis [15]. Histologically, the neoplasm may resemble a sarcoma due to the spindle cells. In contrast to a carcinomasarcoma, which consists of a mixture of glandular and spindle cell differentiation, an undifferentiated carcinoma, sarcomatoid variant, has only a prominent spindle cell differentiation. Cytokeratin is expressed in over 80%. Sarcomatoid carcinomas can be differentiated from carcinosarcomas which immunohistochemically show cytokeratin and vimentin reactivity and are, therefore, not considered to be true carcinomas [16]. The spindle cells of sarcomatoid carcinomas often label for actin, although desmin is not frequently expressed. In our case desmin was expressed and actin was negative.

## Conclusion

In light of these factors, complete resection offers the only potentially curative treatment option in malignant tumors of the head of the pancreas. A preoperative diagnosis could not be obtained in any of our cases without a biopsy. Although adenocarcinoma of the pancreas was suspected preoperatively in each case, the final pathology demonstrated unexpected and unusual diagnoses. Due to the suspicion of a carcinoma, partial pancreaticoduodenectomy was justified in all three patients. Although the Whipple procedure is still considered to be a major surgical intervention with high morbidity and low long-term survival, partial pancreaticoduodenectomy is indicated for all suspicious tumor-like lesions of the head of the pancreas. These unexpected pathologic diagnoses underscore the fact that confirmatory evidence of suspicious diagnoses by surgery is still the gold standard.

## Abbreviations

CT: Computed Tomography; ERCP: Endoscopic Retrograde Cholangiopancreatography; CK: cytokeratin; CD: Cluster of differentiation; CA19-9: carbohydrate antigen 19-9; POD: postoperative day; PPPD: pylorus-preserving pancreaticoduodenectomy.

## Competing interests

The authors declare that they have no competing interests.

## Consent

Written informed consent was obtained from all patients for publication of this case report and accompanying images. A copy of the written consents is available for review by the Editor-in-Chief of this journal.

## Authors' contributions

NL was involved in the conception and design and wrote the first draft of the manuscript. KC was involved in the preparation and review of the manuscript, the literature review and made a substantial intellectual contribution. SEB performed the histopathological work-up and contributed to the pathology part of the manuscript. PK performed the radiological work-up and contributed to the radiology part of the manuscript. JSaE and CFE performed surgery and critically revised the manuscript. WTK was involved in the initiation of the report and made a substantial intellectual contribution to the conception, acquisition and interpretation of the data. All authors read and approved the final manuscript.
